# Tyrosine kinase inhibitors for solid tumors in the past 20 years (2001–2020)

**DOI:** 10.1186/s13045-020-00977-0

**Published:** 2020-10-27

**Authors:** Liling Huang, Shiyu Jiang, Yuankai Shi

**Affiliations:** Department of Medical Oncology, National Cancer Center/National Clinical Research Center for Cancer/Cancer Hospital, Chinese Academy of Medical Sciences & Peking Union Medical College, Beijing Key Laboratory of Clinical Study On Anticancer Molecular Targeted Drugs, No. 17 Panjiayuan Nanli, Chaoyang District, Beijing, 100021 China

**Keywords:** Tyrosine kinase inhibitors, Solid tumors, Targeted therapy

## Abstract

Tyrosine kinases are implicated in tumorigenesis and progression, and have emerged as major targets for drug discovery. Tyrosine kinase inhibitors (TKIs) inhibit corresponding kinases from phosphorylating tyrosine residues of their substrates and then block the activation of downstream signaling pathways. Over the past 20 years, multiple robust and well-tolerated TKIs with single or multiple targets including EGFR, ALK, ROS1, HER2, NTRK, VEGFR, RET, MET, MEK, FGFR, PDGFR, and KIT have been developed, contributing to the realization of precision cancer medicine based on individual patient’s genetic alteration features. TKIs have dramatically improved patients’ survival and quality of life, and shifted treatment paradigm of various solid tumors. In this article, we summarized the developing history of TKIs for treatment of solid tumors, aiming to provide up-to-date evidence for clinical decision-making and insight for future studies.

## Introduction

According to GLOBOCAN 2018, an estimated 18.1 million new cancer cases and 9.6 million cancer deaths occurred in 2018 worldwide [1]. Targeted agents are superior to traditional chemotherapeutic ones in selectivity, efficacy, and safety by acting on specific targets involved in proliferation and differentiation of cancer cells with minimal activity on normal cells.

At least 58 receptor tyrosine kinases (RTKs) and 32 non-receptor tyrosine kinases (NRTKs) have been found so far [2]. RTKs and NRTKs function by catalyzing the transfer of a phosphoryl group from a nucleoside triphosphate donor to the hydroxyl group of tyrosine residues on protein substrates and then triggering the activation of downstream signaling cascades [3]. Abnormal activation of tyrosine kinases due to mutations, translocations, or amplifications is implicated in tumorigenesis, progression, invasion, and metastasis of malignancies. In addition, wild-type tyrosine kinases can also function as critical nodes for pathway activation in cancer. As such, tyrosine kinases have emerged as major targets for drug discovery [4, 5]. A tyrosine kinase inhibitor (TKI) is designed to inhibit the corresponding kinase from playing its role of catalyzing phosphorylation [6]. Since US Food and Drug Administration (FDA) approved imatinib for the treatment of chronic myeloid leukemia in 2001, multiple potent and well-tolerated TKIs—targets including EGFR, ALK, ROS1, HER2, NTRK, VEGFR, RET, MET, MEK, FGFR, PDGFR, and KIT—have been emerging and contributing to the significant progress in cancer treatment. Besides TKIs with one target, some TKIs block a broader range of targets, such as VEGFR-associated multi-targeted TKIs. Noted that some of the multi-targeted TKIs were initially designed to be highly selective, but they turned out to cover other unexpected targets as well [7, 8].

**Fig. 1:**
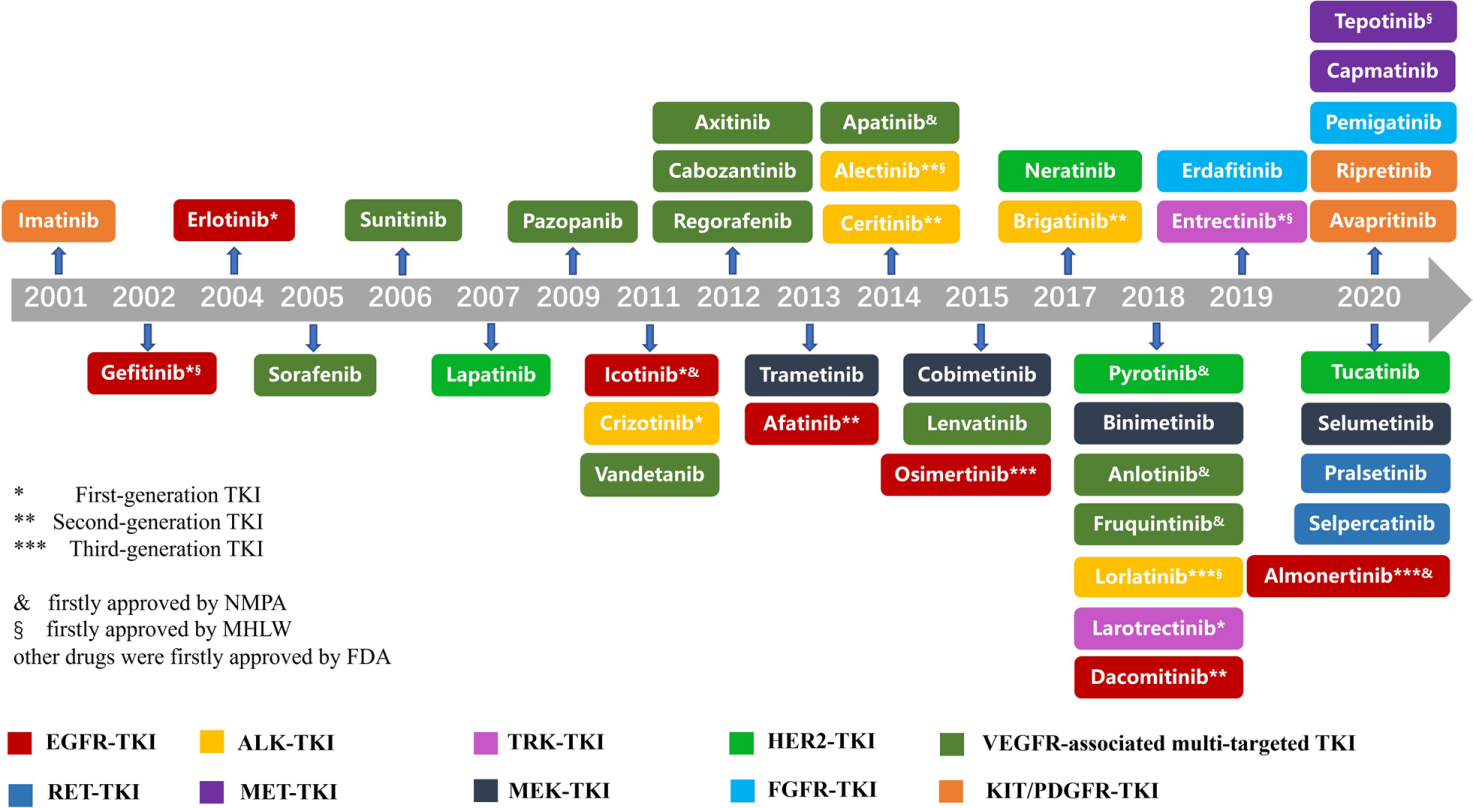
A schematic summary of the approved TKIs in 2001–2020. *NMPA* National Medical Products Administration, *MHLW* Ministry of Health, Labor and Welfare, *FDA* Food and Drug Administration

In this article, we summarized the developing history of TKIs for treatment of solid tumors in the past 20 years (2001–2020). And we presented a schematic summary of the approved TKIs for different targets in Fig. 1.

## EGFR-TKIs

The epidermal growth factor receptor (EGFR), also called HER1, belongs to ErbB family which is composed of four structure-related RTKs: HER1-4. EGFR is a transmembrane glycoprotein with tyrosine kinase activity in its endo-domain. The activation of EGFR can initiate several crucial signal cascades including RAS/RAF/MEK/ERK, PI3K/AKT/mTOR, and STAT pathways [9, 10]. EGFR-sensitizing mutations (i.e., exons 19 deletions and exon 21 L858R substitution) occur in 17.3% of Western and 45.7% of Asian patients with lung adenocarcinoma [11, 12]. To date, EGFR-TKIs are relatively in depth researched with four generations being developed and have been playing irreplaceable roles in the treatment of EGFR-mutant NSCLC patients [13]. Table 1 summarizes advances of EGFR-TKIs.

**Table 1:** Advances of EGFR-TKIs

Drug	Brand name	Manufacturer	Targets	Applications of diseases	Approved years or current phases of clinical trials
Gefitinib	Iressa	AstraZeneca	EGFR-sensitizing mutations	Inoperable or recurrent NSCLC	2002^§^
				1L metastatic EGFR-sensitizing mutant NSCLC	2015 [14, 15]
Erlotinib	Tarceva	OSI/Genentech	EGFR-sensitizing mutations	Locally advanced or metastatic NSCLC after failure of at least one prior chemotherapy regimen	2004 [16]
				1L advanced EGFR-sensitizing mutant NSCLC	2016 [16]
				Combined with ramucirumab for 1L advanced EGFR-sensitizing mutant NSCLC	2020 [191]
Icotinib	-	Shanghai Beta	EGFR-sensitizing mutations	Locally advanced or metastatic NSCLC after failure of at least one prior chemotherapy regimen	2011^&^
				1L metastatic EGFR-sensitizing mutant NSCLC	2014^&^ [17, 18]
Afatinib	Gilotrif	Boehringer Ingelheim	EGFR, HER2	Metastatic EGFR-sensitizing mutant NSCLC	2013 [192–194]
				Advanced SqCC of Lung whose disease has progressed after treatment with platinum-based chemotherapy	2016 [195]
				1L metastatic NSCLC with non-resistant EGFR mutations (L861Q, G719X and S768I)	2018 [192, 196]
Dacomitinib	Vizimpro	Pfizer Inc	EGFR, HER2	1L metastatic EGFR-sensitizing mutant NSCLC	2018 [22, 23]
Osimertinib	Tagrisso	AstraZeneca	EGFR T790M, EGFR-sensitizing mutations	EGFR-T790M NSCLC	2015 [25]
				1L metastatic EGFR-sensitizing mutant NSCLC	2018 [26, 27]
				Metastatic or recurrent NSCLC with EGFR mutations other than the exon 19 deletion, L858R and T790M mutations, and exon 20 insertion	II [44]
Almonertinib	-	Jiangsu Hansoh	EGFR T790M, EGFR-sensitizing mutations	EGFR-T790M NSCLC	2020^&^ [32]
				1L locally advanced or metastatic pulmonary adenosquamous carcinoma	III (NCT04354961)
Investigational drugs					
AST2818 (furmonertinib)	-	Shanghai Allist	EGFR T790M, EGFR-sensitizing mutations	Advanced EGFR-T790M NSCLC	II [33, 34]
				1L locally advanced or metastatic EGFR-sensitizing mutant NSCLC	III (NCT03787992)
YH25448 (lazertinib)	-	Yuhan	EGFR T790M, EGFR-sensitizing mutations	Advanced EGFR-activating mutated NSCLC	I/II [35]
				1L locally advanced or metastatic EGFR-sensitizing mutant NSCLC	III (NCT04248829)
BPI-7711	-	Shanghai Beta	EGFR T790M, EGFR-sensitizing mutations	EGFR-T790M advanced or recurrent NSCLC	I [36] IIb (NCT03812809)
				1L locally advanced or recurrent/metastatic EGFR-sensitizing mutant NSCLC	III (NCT03866499)
EGF816 (nazartinib)	-	Novartis	EGFR T790M, EGFR-sensitizing mutations	Advanced EGFR-mutant NSCLC	I [37]
TAK-788 (mobocertinib)	-	Takeda	EGFR, HER2	Metastatic NSCLC with EGFR exon 20 insertions whose disease has progressed on or after platinum-based chemotherapy	2020/4/27 Breakthrough therapy recognition [45]
				1L locally advanced or metastatic NSCLC with EGFR exon 20 insertions	III (NCT04129502)
Poziotinib	-	Hanmi	EGFR, HER2, HER4	≥ 2L advanced NSCLC with EGFR exon 20 insertions	II [46]
Tarloxotinib	-	Rain	EGFR, HER2, NRG fusion	2L NSCLC with EGFR exon 20 insertion or HER2-activating mutation, solid tumors With NRG1/ERBB family gene fusions	[197] II (NCT03805841)
TQB3804	-	Jiangsu ChiaTai Tianqing	EGFR T790M, C797S, EGFR-sensitizing mutations	Osimertinib-resistant EGFR T790M/C797S mutant NSCLC	[42] I (NCT04128085)
EAI045	-	CSN	L858R, EGFR T790M, C797S	Combined with cetuximab for osimertinib-resistant EGFR T790M/C797S mutant NSCLC	[41]

In the last column of “Approved years or current phases of clinical trials”: if a drug has been approved, we provided data of the year of its approval by Food and Drug Administration (FDA) except gefitinib with a superscript “§” which means it was firstly approved by Ministry of Health, Labor and Welfare (MHLW), and almonertinib with a superscript “&” which means it was approved by National Medical Products Administration (NMPA); if a drug is under investigation, we provided current phases of its clinical trials

*EGFR* epidermal growth factor receptor, *TKIs* tyrosine kinase inhibitors, *EGFR-sensitizing mutations:* EGFR exon 19 deletions or exon 21 L858R substitution mutations, *SqCC* squamous cell carcinoma

Data source: www.fda.gov, www.drugs.com, and www.clinicaltrials.gov (cutoff date: 19 July 2020)

### First- and second-generation EGFR-TKIs for EGFR-sensitizing mutations

First-generation reversible EGFR-TKIs (e.g., gefitinib [14, 15], erlotinib [16] and icotinib [17, 18]) have yielded significant survival benefits for patients with advanced NSCLC harboring EGFR-sensitizing mutations. Additionally, efforts to investigate them in adjuvant setting have also been made [19–21]. Second-generation EGFR-TKIs (e.g., afatinib, dacomitinib) bind irreversibly to EGFR and typically belong to pan-HER inhibitors. Dacomitinib yielded an improved median progression-free survival (mPFS) (14.7 vs 9.2 months; hazard ratio (HR) 0.59; *p* < 0.0001) and median overall survival (mOS) (34.1 vs 26.8 months; HR 0.76; *p* = 0.044) compared to gefitinib in first-line treatment of advanced EGFR-mutant NSCLC [22, 23]. However, both afatinib and dacomitinib have increased toxicities, which may limit their use in clinical practice.

### Third-generation EGFR-TKIs

Approximately 50% of resistance to first- and second-generation EGFR-TKIs are due to EGFR T790M mutation, in which the significantly bulkier methionine residue replaces the small polar threonine at position 790 of EGFR exon 20. As a gatekeeper to the adenosine triphosphate (ATP) binding pocket of EGFR, T790M could cause conformational change resulting in the development of steric hindrance; it could also increase the ATP affinity; all of these reduce binding ability and access of first- and second-generation EGFR inhibitors to the EGFR ATP binding pocket [24].

Osimertinib is a third-generation irreversible EGFR-TKI that inhibits both EGFR-sensitizing and EGFR T790M mutations, and was initially approved for NSCLC with EGFR T790M mutation [25]. Later, it also demonstrated superiority over gefitinib or erlotinib in the first-line treatment of EGFR-mutant NSCLC [26, 27]. Along with its favorable safety, osimertinib is likely to surpass other approaches in the standard of care [28]. In addition, osimertinib as adjuvant therapy for stage IB–IIIA EGFR-mutant NSCLC after complete tumor resection also achieved meaningful survival results [29].

Up to 40% of NSCLC patients with EGFR mutation develop central nervous system (CNS) metastases on or after first- or second-generation EGFR-TKIs treatment due to their poor penetration of the blood–brain barrier (BBB). Notably, osimertinib demonstrated favorable efficacy for patients harboring CNS metastases with or without prior EGFR-TKIs treatment [30] or those with EGFR T790M mutation [25]. Besides, EGFR-mutated NSCLC patients with leptomeningeal metastases could also benefit from osimertinib after progression on previous EGFR-TKIs [31].

Almonertinib, another third-generation EGFR-TKI, was approved by China National Medical Products Administration (NMPA). In its phase II trial, an objective response rate (ORR) of 68.9% was observed in patients with previously treated EGFR T790M-positive NSCLC along with a CNS ORR of 60.9% [32]. Other third-generation EGFR-TKIs including furmonertinib (AST2818) [33, 34], lazertinib (YH25448) [35], BPI-7711 [36], and nazartinib (EGF816) [37] have shown promising efficacies and acceptable safeties in advanced NSCLC with EGFR T790M mutation. In a phase IIb trial of furmonertinib, an ORR of 73.6% was observed in patients with EGFR T790M mutated NSCLC [34]. In safety analysis, skin and gastrointestinal disorders as well as interstitial lung disease (ILD) related to furmonertinib seem to be less common than osimertinib [33].

### Strategies for resistance of osimertinib

Long-term responses of third-generation EGFR-TKIs are often compromised by acquired resistant mutations, with EGFR exon 20 C797S mutation as the predominant cause [10]. The prime therapeutic strategy after resistance of osimertinib remains unclear. Though patients harboring C797S in trans with T790M (at different alleles) may respond to the combination of first- and third-generation EGFR-TKIs [38, 39], patients harboring C797S in cis with T790M (at the same allele), which is more common, are likely to show no response [40]. Fourth-generation EGFR-TKIs are under development to overcome osimertinib resistance mediated by EGFR-dependent mutation mechanisms, such as EAI045 against T790M and C797S mutations [41], and TQB3804 against osimertinib-resistant EGFR triple mutant (d746-750/T790M/C797S, L858R/T790M/C797S) or double mutant (d746-750/T790M, L858R/T790M) [42]. In addition, the combination of brigatinib with cetuximab has showed preliminary efficacy in patients with EGFR/T790M/cis-C797S triple mutation [43].

### TKIs for rare EGFR mutations

Targeted therapy for rare EGFR mutations remains an unmet need in NSCLC. Osimertinib showed efficacy against NSCLC with uncommon mutations including L861Q, G719X, or S768I substitutions [44]. Several agents such as TAK-788 (mobocertinib), poziotinib, and tarloxotinib are under investigations for a refractory variant: EGFR exon 20 insertion. A phase I/II study of TAK-788 demonstrated an ORR of 54% in previously treated NSCLC patients harboring EGFR exon 20 insertions [45] and was granted a breakthrough therapy designation by FDA. Poziotinib failed to meet its primary endpoint (ORR 14.8%), but induced tumor reduction in 65% of NSCLC patients with EGFR exon 20 insertion mutants in a phase II trial [46].

### Summary

EGFR-TKIs are effective treatment approaches for EGFR-sensiting-mutant NSCLC. T790M mutation has been the most common mechanism of resistance to first- or second-generation EGFR-TKIs, which fortunately can benefit from third-generation EGFR-TKIs. Novel inhibitors for uncommon EGFR mutations have been emerging. Besides, fourth-generation EGFR-TKIs are under development for resistance of third-generation ones caused by C797S mutation. Moreover, combination treatments have been under investigations. Unlike the concern of toxicities with EGFR-TKIs in combination with the programmed death ligand 1 (PD-L1) antibody [47], combining EGFR-TKIs with anti-VEGF antibody (e.g., ramucirumab) or chemotherapy has shown survival benefit in patients with EGFR mutations.

## ALK-TKIs

The fusion gene of echinoderm microtubule-associated protein-like 4 and anaplastic lymphocyte kinase (EML4-ALK) occurs in 3–5% of NSCLC [48]. It is called “diamond mutation” for the exceedingly prolonged survival benefit from sequential treatment of potent ALK-TKIs [49].

### First- and second-generation ALK-TKIs

First-generation ALK-TKI crizotinib, which targets ALK, ROS1, and c-MET, showed superiority for ALK-positive NSCLC over chemotherapy [50]. However, its unsatisfactory PFS benefits and limited control of brain metastases pushed the development of second-generation ALK-TKIs (ceritinib, alectinib and brigatinib) which are characterized as higher selectivity and CNS penetration, and they are generally effective after failure of crizotinib [51]. As a new ALK-TKI, ensartinib potently inhibits wild-type ALK and common crizotinib-resistant mutations, demonstrating an ORR of 52% in patients who were progressed on crizotinib [52, 53].

Second-generation ALK-TKIs have shown favorable efficacies after progression on crizotinib in clinical practice. A mOS of 89.6 months has been reported in 84 ALK-positive NSCLC patients by the sequencing treatment of second-generation ALK-TKIs after crizotinib resistance in a real-world setting [54]. Moreover, attempt to set second-generation ALK-TKIs as first-line therapy in ALK-positive NSCLC has achieved surprising outcomes, and its standard place has been established. Alectinib is considered as a preferred choice, while ceritinib and brigatinib can serve as other recommended options. In a phase III study of alectinib, it significantly prolonged mPFS compared to crizotinib in treatment-naïve advanced ALK-positive NSCLC patients (34.8 vs 10.9 months; HR 0.43) [55, 56]. Despite the superiority of ceritinib over crizotinib predicted by adjusted indirect comparison in front-line setting [57], no comparative study has been prospectively conducted yet. For brigatinib, a superior mPFS over crizotinib was also observed in the first-line setting (24.0 vs 11.0 months; HR 0.49; *p* < 0.0001) [58, 59]. It has received an approval from both European Medicines Agency (EMA) and FDA as a first-line approach for metastatic ALK-positive NSCLC. Second-generation ALK-TKIs such as alectinib and brigatinib also demonstrated CNS benefits over crizotinib [59, 60]. Other second-generation ALK-TKIs including WX-0593 [61] and CT707 [62] are under clinical investigations and have produced promising outcomes.

### Third-generation ALK-TKIs

Generally, 56% of patients treated with second-generation ALK-TKIs develop acquired resistance due to secondary ALK mutations [63]. When such resistance occurs, lorlatinib, a third-generation ALK/ ROS1-TKI with potency against most known ALK mutations, is a therapeutic option. In a phase II trial, lorlatinib demonstrated meaningful activity as both first-line and subsequent therapies for ALK-rearranged NSCLC, with an ORR of 90% in treatment-naïve patients, 47% in patients with one previous ALK-TKI treatment, and 38.7% in patients with two or more previous ALK-TKIs treatment [64]. In respect of safety profile, apart from common adverse events (AEs) such as hypercholesterolemia and hypertriglyceridemia, grade ≥ 3 neurological toxicity including peripheral neuropathy (2%), cognitive effect (1%), and dizziness (1%) should be taken with caution. With complex mechanisms of resistance to lorlatinib being identified, future tailored approaches for such patients are warranted [65]. Another third-generation ALK-TKI CT-3505 is under investigation (ChiCTR1900025619).

### Summary

Treatment strategies for ALK-rearranged NSCLC patients have advanced considerably with the development of crizotinib and newer generations of ALK-TKIs. Acquisition of resistance to ALK-TKIs ultimately occurs; the best sequencing strategy of first- to third-generation ALK-TKIs warrants further investigations. Additionally, combination treatment of ALK-TKIs and immune checkpoint inhibitors (ICIs) is associated with higher morbidity in many cases, combination with other classes of agents are ongoing.

## ROS1-TKIs

ROS proto-oncogene 1 (ROS1) rearrangements are identified in 1–2% of NSCLC and in several other malignancies such as cholangiocarcinoma (CCA), glioblastoma, or colorectal cancer [66]. Since kinase domains of ROS1 and ALK share similar amino acid residues, crizotinib, ceritinib, and lorlatinib have also shown clinical benefits in NSCLC patients with ROS1 fusion. Crizotinib and entrectinib are the two recommended first-line options. Crizotinib was approved with an ORR of 72% [67]. Entrectinib was simultaneously approved for the treatment of ROS1-rearranged metastatic NSCLC and NTRK gene fusion-positive solid tumors. In ROS1 fusion-positive NSCLC patients, entrectinib demonstrated an ORR of 77% and an intracranial ORR of 55%, along with an acceptable safety profile [68].

When resistance to first-line therapies occurs, lorlatinib and repotrectinib are alternative options. Lorlatinib demonstrated clinical activity in advanced NSCLC patients with ROS1 fusion, including those who are ROS1-TKI-naïve (ORR 62%), crizotinib pretreated (35%), and those with CNS metastases (first-line ORR 64%; second line 50%) [69]. In a phase I study, repotrectinib showed activity both in ROS1-TKI-naïve (ORR 82%) and ROS1-TKI-treated patients (39%) [70]. DS-6051b is potent against ROS1 G2032R (a predominant mechanism of resistance to crizotinib [71]) and other crizotinib-resistant ROS1 mutations and has demonstrated clinical efficacy [72, 73]. Table 2 summarizes advances of ALK/ROS1-TKIs.

**Table 2:** Advances of ALK/ROS1-TKIs

Drug	Brand name	Manufacturer	Targets	Applications of diseases	Approved years or current phases of clinical trials
Crizotinib	Xalkori	Pfizer Inc	ALK, ROS1, MET	Locally advanced or metastatic ALK + NSCLC	2011 [50]
				Metastatic ROS1 + NSCLC	2016 [67]
				Advanced METex14 + or MET-amplified NSCLC	ongoing [134, 135]
Alectinib	Alecensa	Genentech Inc	ALK	Unresectable, advanced or recurrent ALK + NSCLC	2014^§^
				2L metastatic ALK + NSCLC, 1L metastatic ALK + NSCLC	2015, 2017 [55, 56]
Ceritinib	Zykardia	Novartis	ALK, ROS1	2L metastatic ALK + NSCLC	2014
				1L metastatic ALK + NSCLC	2017 [198]
				Advanced ROS1 + NSCLC	II [199]
Brigatinib	Alunbrig	Takeda	ALK	2L metastatic ALK + NSCLC	2017
				1L metastatic ALK + NSCLC	2020 [58, 59]
				Combined with cetuximab for EGFR/T790M/cis-C797S NSCLC	[43]
Ensartinib	-	Beta/Xcovery Holdings Inc	ALK	2L metastatic ALK + NSCLC	2019 a priority review by NMPA [52]
Entrectinib	Rozlytrek	Genentech Inc	ROS1, TRK, ALK	Metastatic ROS1 + NSCLC	2019 [68]
				TRK fusion + solid tumors	2019 [200]
Lorlatinib	Lorbrena	Pfizer Inc	ALK, ROS1	2-3L metastatic ALK + NSCLC	2018^§^ [64]
Investigational drugs					
Repotrectinib	-	Turning Point	ROS1, TRK, ALK	Advanced ROS1 + NSCLC	I [70]
DS-6051b	-	Daiichi Sankyo	ROS1, TRK	ROS1 + NSCLC	I [72]
				TRK + /ROS1 + solid tumors	I [201]
WX-0593	-	Qilun	ALK, ROS1	ALK + or ROS1+ NSCLC	I [61]
CT-707	-	Centaurus	ALK, FAK, Pyk2	2L advanced ALK + NSCLC	I [62]
CT-3505	-	Shouyao Holdings	ALK	ALK + NSCLC	I (ChiCTR1900025619)

In the last column of “Approved years or current phases of clinical trials”, if a drug has been approved, we provided data of the year of its approval by Food and Drug Administration (FDA), except alectinib and lorlatinib with a superscript “§” which means they were firstly approved by Ministry of Health, Labor and Welfare (MHLW); if a drug is under investigation, we provided current phases of its clinical trials

*FAK* focal adhesion kinase, *Pyk2* proline-rich tyrosine kinase-2, *EMA* European Medicines Agency, *NMPA* National Medical Products Administration, *ALK* anaplastic lymphocyte kinase, *ROS1* ROS proto-oncogene 1

Data source: www.fda.gov, www.drugs.com, and www.clinicaltrials.gov (cutoff date: 19 July 2020)

## HER2-TKIs

Human epidermal growth factor receptor 2 (HER2) is a member of ErbB family and shares similar structure with EGFR. Positive-HER2 was reported in 15–20% of invasive breast cancer and considered to be associated with poor differentiation, rapid cell proliferation, lymph node involvement, and resistance to certain types of chemotherapy [74, 75]. The outcome of patients with HER2-positive breast cancer has been significantly improved in the era of targeted therapy. Four TKIs are available for HER2-positive metastatic breast cancer (MBC), namely lapatinib, neratinib, pyrotinib, and tucatinib. Table 3 summarizes advances of HER2-TKIs.

**Table 3:** Advances of HER2-TKIs

Drug	Brand name	Manufacturer	Targets	Applications of diseases	Approved years or current phases of clinical trials
Lapatinib	Tykerb	GlaxoSmithKline	EGFR, HER2	Combined with capecitabine for HER2-overexpressed metastatic breast cancer who has received prior therapy including an anthracycline, a taxane, and trastuzumab	2007 [76]
				Combined with letrozole for 1L postmenopausal HER2-overexpressed and HR + metastatic breast cancer	2010 [78]
Neratinib	Nerlynx	Puma Biotechnology Inc	EGFR, HER2, HER4	Extended adjuvant treatment for patients with early-stage HER2 + breast cancer	2017 [79]
				Combined with capecitabine for HER2 + metastatic breast cancer who has received two or more prior anti-HER2 based regimens in the metastatic setting	2020 [80]
Pyrotinib	-	Jiangsu Hengrui	EGFR, HER2, HER4	Combination with capecitabine for HER2-positive metastatic breast cancer	2018^&^ [81, 82]
				HER2 exon 20 mutant advanced NSCLC	NCT02535507 [202]
Tucatinib	Tukysa	Seattle Genetics	HER2	Combination with trastuzumab and capecitabine for unresectable or metastatic HER2-Positive breast cancer	2020 [84]

In the last column of “Approved years or current phases of clinical trials”, we provided data of the approved years by Food and Drug Administration (FDA), except pyrotinib with a superscript “&” which means it was approved by National Medical Products Administration (NMPA)

Data source: www.fda.gov, www.drugs.com, and www.clinicaltrials.gov (cutoff date: 19 July 2020)

Lapatinib is a reversible EGFR/HER2-TKI, which was first approved in combination with capecitabine for patients with HER2-positive MBC who have failed trastuzumab-based therapy [76]. Later lapatinib plus letrozole obtained another approval as a first-line therapeutic option for the post-menopausal MBC patients with co-expressing hormone receptors and HER2 [77, 78].

Neratinib is an irreversible pan-ErbB inhibitor. The role of neratinib in conferring synergic effect and overcoming resistance of trastuzumab has been identified [75]. In a phase III trial, one-year neratinib after trastuzumab-based adjuvant treatment for early-stage HER2 positive breast cancer reduced invasive disease-free survival events (116 vs 163 events; HR 0.73; *p* = 0.0083) without increasing risk of toxicity, which established its first approval [79]. Recently, it received its second approval in combination with capecitabine for adult patients with HER2-positive MBC after two or more prior anti-HER2 treatment. A phase III study showed neratinib plus capecitabine significantly reduced the risk of disease progression or death (HR 0.76; *p* = 0.006) and delayed symptomatic CNS metastasis (*p* = 0.043) compared with lapatinib plus capecitabine [80].

Pyrotinib, another irreversible pan-ErbB inhibitor, was approved by NMPA based on a phase II study, in which pyrotinib plus capecitabine combination showed superior efficacy in previously treated HER2-positive MBC patients compared to lapatinib plus capecitabine combination [81]. Subsequently, its phase III trial only recruited HER2-positive MBC patients pretreated with taxane and trastuzumab and showed significantly longer PFS in pyrotinib plus capecitabine group than lapatinib plus capecitabine group (12.5 vs 6.8 months; HR 0.39; *p* < 0.0001) [82]. In addition, pyrotinib also demonstrated promising efficacy in chemotherapy-treated NSCLC patients harboring HER2 exon 20 mutation with an ORR of 30% [83].

Tucatinib, a highly selective HER2-TKI, was approved in combination with trastuzumab and capecitabine for previously treated HER2-positive MBC. The tucatinib combination group revealed improved 1-year PFS (33.1 vs 12.3%; HR 0.54; *p* < 0.001) and 2-year OS (44.9 vs 26.6%; HR 0.66; *p* = 0.005) compared to placebo plus trastuzumab and capecitabine group. In terms of safety, grade ≥ 3 diarrhea (12.9 vs 8.6%), elevated alanine aminotransferase (ALT) (5.4 vs 0.5%), and elevated aspartate aminotransferase (AST) (4.5 vs 0.5%) were more common in the tucatinib combination group [84].

Brain metastases occur in 30–50% of HER2-positive MBC, which is tricky with limited evidence-based therapeutic options. Though efficacy of HER2-TKI as a single agent was moderate, combinations of HER2-TKI with capecitabine offered survival benefit for HER2-positive patients with brain metastasis [85, 86]. Lapatinib, neratinib, and tucatinib all had successful attempt in this area [87–89].

Intrinsic and acquired resistance of HER2-TKIs has been investigated. Several genes and pathways (including EGFR family, PI3K/Akt/mTOR, RAS/RAF/MEK/MAPK, autophagy, tumor metabolism, low PTEN, PIK3CA mutations, etc.) have been reported to be associated with lapatinib resistance [90] and may provide inspiration for future HER2-TKI development.

## TRK inhibitors

Tropomyosin receptor kinase (TRK) refers to the neurotrophin receptor tyrosine kinase genes NTRK1/2/3 and their respective encoding neurotrophin protein receptors TRKA/B/C. TRK signal pathways play crucial roles in neuronal development and differentiation. Fusions involving NTRK1/2/3 are the most common mechanisms of oncogenic TRK activation, which are found across a wide variety of malignancies independent of tumor lineage and patients’ age. Rare tumors are reported to have a higher TRK fusion frequency than common tumors [91, 92]. TRK inhibition provides a prime example of the basket trial for targeted therapy, wherein same genomic-altered cancers are treated with one matched therapeutic agent regardless of tumor histology [92–94].

### First-generation TRK inhibitors

Larotrectinib and entrectinib are two approved first-generation TRK inhibitors for adult and pediatric (12 years of age and older) patients with solid tumors harboring NTRK gene fusions which are unresectable, resistant, or lack of satisfactory standard therapy.

Larotrectinib is a selective inhibitor of TRKA/B/C which obtained its approval based on the combined analysis of three phase I/II trials involving 17 unique TRK fusion-positive tumor types [95]. In an expanded data set, patients treated with larotrectinib achieved an ORR of 79%, with manageable toxicities: the most common grade 3–4 TRAEs included increased ALT (3%), anemia (2%), and decreased neutrophil count (2%) [96]. Later analysis showed patients who had received more lines of treatment tend to have less effective response to larotrectinib; response rate dropped more sharply as the Eastern Cooperative Oncology Group (ECOG) performance status (PS) got worse, but a ECOG PS score of 1–2 still can benefit from larotrectinib treatment [97].

Entrectinib is a multi-kinase inhibitor targeting TRK, ROS1, and ALK. The pooled analysis revealed an ORR of 57.4% in patients with TRK fusion across 10 tumor types [98]. In a recent study, entrectinib produced favorable responses in children and adolescents with refractory CNS and extracranial solid tumors harboring NTRK, ROS1, or ALK fusions, as well as those with ALK-mutated neuroblastoma [99].

Other multi-kinase inhibitors including crizotinib, cabozantinib, ponatinib, nintedanib, lestaurtinib, altiratinib, foretinib, merestinib, MGCD516, PLX7486, DS-6051b, and TSR-011 have varying degrees of activity against TRKA/B/C in vitro, but their clinical activities have not been as robust as those of larotrectinib and entrectinib [93, 100].

### Second-generation TRK inhibitors

On-target or off-target mechanisms would disappointedly result in resistance to first-generation TRK inhibitors. On-target resistance mechanisms mainly refer to NTRK kinase domain mutations involving amino acid substitutions of the solvent front, the gatekeeper residue, or the xDFG motif [100].

Two major developing second-generation TRK inhibitors selitrectinib (LOXO-195) and repotrectinib (TPX0005) are designed to overcome the acquired on-target resistance of first-generation ones and possess enhanced activities against wild-type TRKA/B/C. Selitrectinib (LOXO-195) selectively targets multiple TRK kinase domain mutations including solvent front and xDFG substitutions [101]. The largest data set of LOXO-195 till now enrolled 31 TRK-fusion patients with 11 cancer types progressing or being intolerant to at least one prior TRK inhibitor: the ORR was 54% in patients with on-target TRK mutations [102]. Repotrectinib (TPX-0005), another next-generation ROS1/TRK/ALK inhibitor, has shown promising anti-tumor activity, a confirmed partial response (reduced by 82%) in an entrectinib-resistant patient with a salivary gland tumor and a tumor regression (reduced by 33%) in a patient with larotrectinib-resistant cholangiocarcinoma were reported [103].

Mechanisms of off-target resistance to first- or second-generation TRK inhibitors include KRAS and BRAF V600E mutations, MET amplifications, IGF1R activation, etc. The convergent activation of mitogen-activated protein kinase (MAPK) pathway was also proposed to mediate the resistance of TRK inhibition. Second-generation TRK inhibitors are ineffective against off-target resistance, whereas a single targeted agent for off-target mutation or combined with TRK inhibition might re-established disease control in this situation. For example, simultaneous inhibition of TRK and MEK (belongs to MAPK pathway) was found to successfully manage some off-target TRK resistance in vitro and vivo [104, 105].

Table 4 summarizes advances of TRK inhibitors.

**Table 4:** Advances of TRK inhibitors

Drug	Brand name	Manufacturer	Targets	Applications of diseases	Approved years or current phases of clinical trials
Larotrectinib	Vitrakvi	Lilly’s Loxo Oncology Inc	TRK	TRK fusion + solid tumors	2018 [95, 96]
Entrectinib	Rozlytrek	Genentech Inc	TRK, ROS1, ALK	Advanced ROS1 + NSCLC	2019^§^ [68, 200]
				TRK fusion + solid tumors	2019^§^ [200]
Investigational drugs					
Selitrectinib (LOXO-195)	-	Lilly’s Loxo Oncology Inc	TRK, most resistant TRK mutations	TRK fusion + solid tumors	I [102]
Repotrectinib (TPX-0005)	-	Turning Point	TRK, ROS1, ALK, most resistant TRK mutations	ROS1, NTRK, or ALK fusion gene fusion solid tumors	[103] I/II (NCT03093116), I/II (NCT04094610)

In the last column of “Approved years or current phases of clinical trials”: larotrectinib was firstly approved by Food and Drug Administration (FDA), entrectinib was firstly approved by Ministry of Health, Labor and Welfare (MHLW), other two drugs are under investigation, we provided current phases of their clinical trials

*TRK* Tropomyosin receptor kinase, *NSCLC* non-small cell lung cancer

Data source: www.fda.gov, www.drugs.com, and www.clinicaltrials.gov (cutoff date: 19 July 2020)

## VEGFR-associated multi-targeted TKIs

The vascular endothelial growth factor (VEGF) family is composed of VEGF-A/B/C/D/E and placental growth factor. VEGF-A, also called VEGF, is the master regulator of angiogenesis. The binding of VEGF to VEGFR-2 plays a key role in stimulating the proliferation and migration of endothelial cells as well as regulating vascular permeability [106]. In recent years, VEGFR-associated multi-targeted TKIs have emerged as potent anti-tumor weapons against multiple solid tumors [107].

### Applications of VEGFR-associated multi-targeted TKIs in hepatocellular carcinoma (HCC)

The potency of VEGFR-associated multi-targeted TKIs was supported by robust evidence in HCC [108]. Sorafenib, targeting VEGFR, PDGFR, FGFR, and other signaling targets, is recommended for front-line therapy for unresectable HCC [109, 110]. When compared with sorafenib, lenvatinib demonstrated a superior mPFS (7.4 vs 3.7 months; *p* < 0.0001) and a non-inferior mOS (13.6 vs 12.3 months; HR 0.92). Lenvatinib produced fewer grade ≥ 3 palmar–plantar erythrodysaesthesia but with higher incidence of hypertension and proteinuria [111]. More recently, the combination of lenvatinib with anti-programmed cell death protein-1 (PD-1) antibody pembrolizumab showed encouraging anti-tumor activity in patients with untreated/unresectable HCC with an ORR of 36%, a mPFS of 8.6 months, and a mOS of 22.0 months [112]. This combination has been granted a breakthrough therapy designation by FDA. In a phase II/III trial involving advanced HCC, another VEGFR-associated multi-targeted TKI donatinib achieved a superior OS over sorafenib (12.1 vs 10.3 months; HR 0.83; *p* = 0.0363) [113]. In addition, several other VEGFR-associated multi-targeted TKIs including regorafenib [114], cabozantinib [115], and apatinib [116] are applied as subsequent-line therapies of HCC.

### Applications of VEGFR-associated multi-targeted TKIs in renal cell carcinoma (RCC), lung cancer, and other cancer type

RCC is another cancer type deriving great benefit from VEGFR-associated multi-targeted TKIs. Sorafenib, sunitinib, pazopanib, cabozantinib, the combination of axitinib and pembrolizumab/avelumab were successively approved as first-line treatment options for metastatic RCC.

Anlotinib has yielded favorable outcomes in lung cancer and was approved for third-line or further-line therapy for both NSCLC [117] and SCLC [118] by NMPA. In its phase II trial for patients with relapsed SCLC, a mPFS of 4.1 months was reported [119]. Similarly, apatinib presented a mPFS of 5.4 months in patients with extensive-stage SCLC after failure of two or more lines of chemotherapy [120].

Furthermore, VEGFR-associated multi-targeted TKIs also demonstrated survival benefits in patients with thyroid carcinoma, soft tissue sarcoma (STS), and other solid malignancies [121] (see Table 5).

**Table 5:** Advances of VEGFR-associated multi-targeted TKIs

Drug	Brand name	Manufacturer	Targets	Applications of diseases	Approved years or current phases of clinical trials
Sorafenib	Nexavar	Bayer	VEGFR1-3, TIE2, PDGFR, FGFR, BRAF, CRAF, KIT, FLT-3	mRCC	2005 [203]
				Unresectable HCC	2007 [109, 110]
				metastatic DTC	2013 [204]
Sunitinib	Sutent	Pfizer Inc	VEGFR-1–2, PDGFR, FLT3, KIT	GIST after disease progression on or intolerance to imatinib	2006 [172]
				mRCC	2006 [172]
				unresectable or metastatic pancreatic neuroendocrine tumor	2011 [205]
				Adjuvant treatment for RCC	2017
Vandetanib	Caprelsa	AstraZeneca	EGFR, VEGFR2-3, RET	Unresectable or metastatic MTC	2011 [206]
Regorafenib	Stivarga	Bayer	VEGFR1-3, TIE2, PDGFR, FGFR, BRAF, KIT, RET	Recurrent or metastatic CRC, locally advanced/unresectable or metastatic GIST previously treated with imatinib and sunitinib, advanced HCC who has been previously treated with sorafenib	2012 [207] , 2013 [173], 2017 [114]
Lenvatinib	Lenvima	Eisai Inc	VEGFR1-3, PDGFR, FGFR 1–4, RET, KIT	Radioactive iodine refractory DTC	2015 [208]
				2L combined with everolimus for mRCC	2016 [209]
				1L unresectable HCC	2018 [111]
				Combined with pembrolizumab for certain types of endometrial cancer	2019 [210]
Cabozantinib	Cometriq/Cabometyx	Exelixis Inc	VEGFR1-3, MET, ROS1, RET, AXL, NTRK, KIT	Progressive metastatic MTC	2012 [211]
				2L mRCC	2016 [212]
				1L mRCC	2017
				HCC who has been previously treated with sorafenib	2019 [115]
Axitinib	Inlyta	Pfizer Inc	VEGFR1-3, PDGFR, KIT, FLT-3	2L advanced RCC	2012 [213]
				1L combined with pembrolizumab for advanced RCC	2019 [214]
				1L combined with avalumab for advanced RCC	2019 [215]
Pazopanib	Votrient	GlaxoSmithKline	VEGFR, PDGFR, KIT	Advanced RCC	2009
				Advanced STS who has previously received chemotherapy	2012
Anlotinib	-	Jiangsu ChiaTai Tianqing	VEGFR2-3, FGFR1-4, PDGFR, KIT, RET	≥ 3L metastatic NSCLC	2018^&^ [117]
				≥ 2L metastatic STS	2018^&^ [216]
				≥ 3L relapsed SCLC	2019^&^ [118]
				Many other solid tumors	ongoing
Fruquintinib	-	Chi-Med	VEGFR1-3	≥ 3L mCRC	2018^&^ [217]
Apatinib	-	Jiangsu Hengrui	VEGFR2, KIT, RET, c-Src	≥3L adenocarcinoma of the stomach or gastroesophageal junction	2014^&^ [218]
				2L HCC	2020^&^ Applying for approval [116]
Investigational drugs					
Surufatinib	-	Chi-Med	VEGFR1-3, FGFR, CSF-1R	1L non-pancreatic NET	2019^&^ Received a NDA [219]
				Pancreatic NET, solid tumors, biliary tract cancer	I-III ongoing
Famitinib	-	Jiangsu Hengrui	VEGFR2-3, PDGFR, FLT1, FLT3, KIT	CRC, NSCLC	III ongoing
				Multiple solid tumors	I-II ongoing
Donafenib	-	Suzhou Zelgen	VEGFR, PDGFR	HCC, CRC, DTC, NPC	I-III ongoing
Cediranib	-	AstraZeneca	VEGFR1-3, KIT, PDGFR	Combined with olaparib for ≥ 2L SCLC	II [220]

In the last column of “Approval years or current phases of clinical trials”, if a drug has been approved, we provided data of the year of its approval by Food and Drug Administration (FDA), except those with a superscript “&” which means they were firstly approved by National Medical Products Administration (NMPA); if a drug is under investigation, we provided current phases of its clinical trials

*NSCLC* non-small cell lung cancer, *SCLC* small cell lung cancer, *mRCC* metastatic renal cell carcinoma, *HCC* hepatocellular carcinoma, *DTC* differentiated thyroid cancer, *MTC* medullary thyroid cancer, *CRC* colorectal cancer, *GIST* gastrointestinal stromal tumor, *STS* soft tissue sarcoma, *NPC* nasopharyngeal carcinoma, *NET* neuroendocrine tumors, *FLT3* fetal liver tyrosine kinase receptor 3, *NDA* new drug application, *VEGFR* vascular endothelial growth factor receptor

Data source: www.fda.gov, www.drugs.com, and www.clinicaltrials.gov (cutoff date: 19 July 2020)

### Anti-angiogenesis and PD-1/PD-L1 inhibition

Preclinical and clinical studies suggested that the combination of anti-angiogenesis inhibitors with ICIs could provide superior anti-tumor activity over either single agent. VEGFR inhibitors might potentially improve immunotherapeutic activity of PD-1/PD-L1 antibodies by enhancing tumor infiltration of immune cells and reducing immunosuppressive effects of myeloid-derived suppressor cells [122]. Investigational combinations of VEGFR-associated multi-targeted TKIs and anti-PD-1/PD-L1 antibodies are summarized in Table 6.

**Table 6:** Investigational combinations of VEGFR-associated multi-targeted TKIs and anti-PD-1/PD-L1 antibodies

Combination therapy	Application	Clinical trial	Publications
Lenvatinib + pembrolizumab	Unresectable HCC	KEYNOTE-524/Study116 (Ib, NCT03006926) LEAP-002 (III, NCT03713593)	[112]
	Solid tumors	Ib/II, NCT02501096	Ongoing [221]
Apatinib + camrelizumab	2L SCLC	PASSION (II, NCT03417895)	[222]
Axitinib + pembrolizumab	1L RCC	KEYNOTE426 (III, NCT02853331)	[214]
Axitinib + avelumab	1L RCC	JAVELIN Renal 101 trial (III, NCT02684006)	[215, 223]
Axitinib + toripalimab	1L metastatic mucosal melanoma	Ib (NCT03086174)	[224]
Regorafenib + avelumab	Non-MSI-H mCRC	I/II (NCT03475953)	[225]
Surufatinib + toripalimab	Solid tumors	I (NCT03879057)	[226]

*HCC* hepatocellular carcinoma, *SCLC* small cell lung cancer, *RCC* renal cell carcinoma, *Non-MSI-H* non-microsatellite instability-high

Data source: www.fda.gov, and www.clinicaltrials.gov (cutoff date: 19 July 2020)

## RET-TKIs

The rearranged during transfection (RET) tyrosine kinase plays a role in transducing signals involving cell growth and differentiation. RET alterations (i.e., RET fusions and point mutations) are implicated in the pathogenesis of lung, thyroid, and other cancers. RET fusions were found in 10–20% of papillary thyroid cancers and 1–2% of NSCLC, while RET point mutations occur in approximately 60–90% of advanced medullary thyroid cancers (MTC) [123]. Table 7 summarizes advances of RET-TKIs.

**Table 7:** Advances of RET-TKIs

Drug	Brand name	Manufacturer	Targets	Applications of diseases	Approved years or current phases of clinical trials
Selpercatinib (LOXO-292)	Retevmo	Eli Lilly and Company	RET	Metastatic RET fusion-positive NSCLC, advanced or metastatic radioactive iodine-refractory thyroid cancer, advanced or metastatic RET-mutant MTC	2020 [227, 228]
				1L advanced or metastatic RET fusion-positive NSCLC	III(NCT04194944)
				Advanced RET-mutant MTC	III(NCT04211337)
Pralsetinib (BLU-667)	–	Blueprint Medicines	RET	Advanced RET fusion-positive NSCLC	2020 [127]
				RET fusion + solid tumors	I/II [128]
				1L advanced RET fusion-positive NSCLC	III (NCT04222972)

In the last column of “Approved years or current phases of clinical trials”: both drugs were approved by Food and Drug Administration (FDA), and we also provided their current phases of clinical trials

*NDA* new drug application, *NSCLC *non-small cell lung cancer, *MTC* medullary thyroid cancer, *RET* rearranged during transfection

Data source: www.fda.gov, www.drugs.com, and www.clinicaltrials.gov (cutoff date: 19 July 2020)

Before any selective RET inhibitor becomes available, chemotherapy, multi-targeted TKIs, and clinical trials are common choices for RET-altered cancer patients. Multi-targeted TKIs including cabozantinib and vandetanib have been clinically used in RET-driven lung and thyroid cancers, but their insufficient inhibition of RET and off-target toxicities limited broader application [124]. Likewise, NSCLC patients with RET rearrangements have minimal response to immunotherapy (ORR 6%) [112].

Currently, two selective RET-TKIs shed light in this area. Selpercatinib (LOXO-292) is the first approved RET-TKI with applications for advanced RET fusion-positive NSCLC, thyroid cancer, and RET-mutant MTC. In the treatment of RET fusion-positive NSCLCs, selpercatinib presented an ORR of 85% in patient who were systemic treatment-naïve ones, 64% in previously treated patients, 91% in patients with CNS metastases [125]. In the treatment of RET-altered thyroid cancers, the ORRs were 73% and 69%, respectively, in treatment-naïve and previously treated RET-mutant MTCs patients, and 79% in previously treated thyroid cancers patients with RET fusion. In general, selpercatinib was well tolerated, with only 2% of 531 patients discontinuing treatment due to TRAEs [126].

Pralsetinib (BLU-667) recently obtained a rolling new drug application (NDA) submission for RET fusion-positive NSCLC. It demonstrated promising clinical efficacy regardless of RET fusion genotype or prior therapies status. The ORR was 73% for treatment-naïve NSCLC patients harboring RET fusion and 61% for platinum-exposed patients. It is well tolerated with most TRAEs being grade 1–2, including increased AST (31%), anemia (22%), increased ALT (21%), constipation (21%), and hypertension (20%) [127]. A broad range of anti-tumor activity of pralsetinib on multiple advanced RET fusion-positive solid tumors in addition to NSCLC has also been reported; tumor type includes papillary thyroid cancers, undifferentiated thyroid cancer, pancreatic cancer, colon cancer, etc.[128].

Up-to-date evidence of resistance mechanism to selective RET inhibitor remains limited. RET G810 solvent front mutation represents a recurrent mechanism of resistance to selpercatinib and should be considered when developing more potent or next-generation RET-TKIs [129].

## MET-TKIs

The mesenchymal–epithelial transition factor (MET) is also called c-MET or hepatocyte growth factor receptor (HGFR). The binding of MET to its ligand HGF activates various signaling pathways and plays a role in cellular proliferation, motility, migration, and invasion [130–132]. Identifying potential patients sensitive to MET inhibitors by detection of MET exon14 skipping alterations (METex14) or MET amplification has made some progress in recent years. Table 8 summarizes advances of MET-TKIs.

**Table 8:** Advances of MET-TKIs

Drug	Brand name	Manufacturer	Targets	Applications of diseases	Approved years or current phase of clinical trials
Capmatinib	Tabrecta	Novartis Oncology	MET	Metastatic METex14 + NSCLC	2020 [140]
				Combined with gefitinib for MET-amplified EGFR-mutant NSCLC with required EGFR-TKI resistance	Ib/II [148]
Tepotinib	Tepmetko	Merck	MET	Metastatic METex14 + NSCLC	[137, 139] 2020 NDA accepted by FDA 2020 approved by MHLW
				Combined with gefitinib for MET-amplified EGFR-mutant NSCLC with required EGFR-TKI resistance	Ib/II [146]
				Combined with osimertinib for osimertinib relapsed MET-amplified EGFR-mutant NSCLC	II (NCT03940703)
Savolitinib	-	AstraZeneca and Chi-Med	MET	METex14 + PSC and other types of NSCLC Combined with osimertinib for osimertinib relapsed MET-amplified EGFR-mutant NSCLC	II [143] Ib [149] III(NCT03778229)

In the last column of “Approved years or current phases of clinical trials”: capmatinib was firstly approved by FDA; tepotinib was firstly approved by MHLW and also received an NDA from FDA; savolitinib was received a priority review by NMPA for metastatic METex14 + NSCLC. We also provided their current phases of clinical trials

*PSC* pulmonary sarcomatoid carcinoma, *NSCLC* non-small cell lung cancer, *FDA* Food and Drug Administration, *NDA* new drug application, *MHLW* Ministry of Health, Labor and Welfare, *MET* mesenchymal–epithelial transition factor, *METex14* MET exon14 skipping alterations

Data source: www.fda.gov, www.drugs.com, and www.clinicaltrials.gov (cutoff date: 19 July 2020)

### Targeting MET as the primary oncogenic event of NSCLC

METex14 occur in approximately 3% of lung adenocarcinoma, 2% of other lung neoplasms, and rare in other tumors [133]. Intriguingly, crizotinib was initially developed as a MET inhibitor and later on made great achievements in ALK and ROS1 inhibition. But it still showed meaningful activity against MET amplification and METex14 [134]. In a recent study, crizotinib demonstrated an ORR of 32% in NSCLC patients with METex14-mutation [135]. Other multi-targeted TKIs such as cabozantinib, merestinib, glesatinib, and sitravatinib also showed meaningful activities against MET [136].

Apart from multi-targeted TKIs, selective MET inhibitors like tepotinib, camaptinib, and savolitinib have emerged with promising survival benefits. Tepotinib has received a breakthrough therapy designation by FDA for treatment of metastatic NSCLC after failure of platinum-based therapy with an ORR of 47% and a mPFS of 11 months. 27% of patients experienced grade 3–4 AEs, with peripheral edema being the most common AE (7%) [137–139]. Recently, tepotinib was approved by Japanese Ministry of Health, Labor and Welfare (MHLW) for the treatment of unresectable, advanced or recurrent NSCLC with METex14 mutation, making it the first approved MET-TKI worldwide.

Another MET inhibitor, capmatinib, was approved for the treatment of adult NSCLC patients with METex14 mutation regardless of treatment history. In a phase II study, the efficacy of capmatinib was evaluated in advanced NSCLC patients with METex14 mutation or MET amplification across 6 cohorts. The ORRs were 41% and 68% among cohort 4 (previously treated METex14 mutation) and cohort 5b (treatment-naïve METex14 mutation), respectively. Its safety profile was acceptable across all cohorts (n = 315), with peripheral edema (49.2%), nausea (43.2%), and vomiting (28.3%) as the most common AEs [140]. Other cohorts also demonstrated the efficacy of capmatinib in advanced NSCLC with high-level MET amplification [141, 142].

Savolitinib (also called volitinib) demonstrated promising anti-tumor activity and manageable toxicity in pulmonary sarcomatoid carcinoma (PSC) and other types of NSCLC with METex14-mutation, with an ORR of 47.5%. Notably, 14.3% of patients discontinued savolitinib due to TRAEs, with liver injury and hypersensitivity being the most common AEs (each 2.9%) [143].

### Targeting MET as the secondary event of EGFR-TKI resistant NSCLC

MET amplification is an important resistant mechanism of EGFR-TKIs for NSCLC treatment, accounting for 6.25–22%. More importantly, this patient population is unlikely to respond well to osimertinib or other third-generation EGFR-TKIs. Preclinical and clinical data suggest the combination of EGFR-TKIs with MET-TKIs could be an alternative option to overcome MET-driven acquired resistance of NSCLCs who have progressed on a previous EGFR-TKI [144, 145]. For instance, tepotinib plus gefitinib significantly prolonged mPFS (19.3 vs 5.5 months; HR 0.18), mOS (37.3 vs 13.1 months; HR 0.08), and ORR (75.0 vs 42.9%; OR 4.00) compared to chemotherapy for such patient population. In terms of safety, tepotinib plus gefitinib combination treatment significantly increased grade ≥ 3 amylase and lipase, while anemia, neutrophil, or white blood cell count decrease was less common compared to chemotherapy [146]. In a phase Ib/II trial, capmatinib plus gefitinib yielded an ORR of 47% in EGFR-mutant NSCLC patients with high MET amplification. The most common grade 3–4 AEs were also elevated amylase and lipase levels [147, 148]. Now with increasing use of osimertinib in the front-line treatment of EGFR-mutant NSCLC, combining MET-TKIs with osimertinib has also been explored. Savolitinib plus osimertinib presented an ORR of 48% (with or without a previous third-generation EGFR-TKI) along with acceptable tolerability [149]. The exciting results suggest it may be necessary to identify MET status before starting osimertinib treatment in patients who failed previous former-generation EGFR-TKI treatment.

Fluorescence in situ hybridization (FISH), next generation sequencing (NGS), immunohistochemistry (IHC), and droplet digital PCR (ddPCR) are methods to detect MET-mediated resistance, each with its own advantages and disadvantages. The results of different testing methods do not overlap completely, and a single assay might overlook suitable patients. Therefore, applying more than one method is recommended in future clinical practice and scientific research. Besides, other biomarkers like phosphorylated MET (p-MET) should be explored to help predict response and tailor treatment.[144, 149].

## MEK-TKIs

The classic mitogen-activated protein kinase (MAPK) pathway—RAS/RAF/MEK/ERK—is critical in signal transduction, whose dysregulation is implicated in one third of all malignancies. RAS and RAF mutations are implicated in a great portion of malignancies: BRAF V600 mutation is found in 40–60% of melanomas and 10–12% of metastatic colorectal cancer (mCRC), KRAS or NRAS in 55% of mCRC, and KRAS in 20–30% of lung adenocarcinoma [150]. Although the mutation of MEK, also called mitogen-activated protein kinase kinase (MAPKK), is not frequently identified in solid tumors, it is a central and critical component that lies downstream of RAS and RAF, and upstream of ERK for transduction. Currently, MEK serves as a hotspot target for the treatment of RAS/RAF mutant cancers [151].

MEK inhibitors function mainly by non-ATP-competitively blocking the phosphorylation of tyrosine and serine/threonine domain of its downstream ERK [152]. To date, four MEK inhibitors–trametinib, cobimetinib, binimetinib, and selumetinib, as single agent or in combination with BRAF inhibitors–have been approved for melanoma/NSCLC/neurofibromas [153–158]. In the treatment of BRAF V600 mutant melanoma, the combination of MEK and BRAF inhibition achieved better outcome than used alone, with manageable safety profiles and lower rates of hypoproliferative skin toxicities and musculoskeletal complaints than BRAF inhibitors, and has become the standard of therapy [157, 159]. In addition, in the treatment of BRAF V600 mutant mCRC, the addition of binimetinib to the doublet inhibition of BRAF and EGFR (encorafenib and cetuximab) showed promising results in a phase III trial [160].

The advances of MEK-TKIs are summarized in Table 9.

**Table 9:** Advances of MEK-TKIs

Drug	Brand name	Manufacturer	Targets	Applications of diseases	Approved years
Trametinib	Mekinist	GlaxoSmithKline	MEK1/2	Unresectable or metastatic BRAF V600E/K + melanoma	2013 [153]
				Combined with dabrafenib for the same condition above	2014 [154]
				Combined with dabrafenib for metastatic BRAF V600E + NSCLC who received previous treatment with chemotherapy	2017
				Combined with dabrafenib for locally advanced or metastatic BRAF V600E + ATC with no locoregional treatment options	2018 [155]
Cobimetinib	Cotellic	Genentech Inc	MEK1/2	Combined with vemurafenib for BRAF V600E/K + advanced or unresectable melanoma	2015 [156]
Binimetinib	Mektovi	Array BioPharma Inc	MEK1/2	Combined with encorafenib for unresectable or metastatic BRAF V600E/K + melanoma	2018 [157]
Selumetinib	Koselugo	AstraZeneca and Merck	MEK1/2	Pediatric patients (≥ 2 years old) with symptomatic, inoperable NF1 plexiform neurofibromas	2020 [158]

In the last column, the four drugs were firstly approved by Food and Drug Administration (FDA)

*ATC* anaplastic thyroid cancer, *NF1* neurofibromatosis type 1

Data source: www.fda.gov, www.drugs.com

## FGFR-TKIs

The fibroblast growth factor (FGF) pathway is implicated in tumor growth and angiogenesis [161, 162]. Most FGFR-TKIs approved belong to multi-targeted TKIs (Table 5). Meanwhile, several FGFR inhibitors have achieved applications for certain cancers recently, such as erdafitinib for urothelial carcinoma and pemigatinib for CCA.

Mutations and fusions in FGFR2/3 occur in 20% of patients with urothelial carcinoma [163]. The FGFR1-4 TKI erdafitinib has been approved for the treatment of adult patients with previously treated locally advanced or metastatic urothelial carcinoma harboring susceptible FGFR2/3 mutation with an ORR of 40%. The response was more favorable compared to antibody–drug conjugates such as enfortumab vedotin or sacituzumab govitecan (ORRs of 33–34%) [164] and pembrolizumab (20.1%) as second-line therapy for advanced urothelial carcinoma [165]. 59% of patients who had undergone previous immunotherapy responded to erdafitinib treatment. Nevertheless, it should be noted that nearly half of the patients experienced at least one grade ≥ 3 AE, among which hyponatremia (11%), stomatitis (10%), and asthenia (7%) were most common [166]. Other pan-FGFR inhibitors are under development; for example, infigratinib (BGJ 398) has produced an ORR of 25.4% in the treatment of FGFR3-mutated urothelial carcinoma [164].

In addition to urothelial malignancies, FGFR2 alterations are also implicated in CCA. A FGFR1-3 TKI pemigatinib has been approved for the treatment of locally advanced or metastatic CCA harboring FGFR2 fusions or rearrangements with an ORR of 35.5% [167]. Several other FGFR-TKIs (e.g., futibatinib, infigratinib [168]) have shown promising results for CCA.

Table 10 summarizes advances of FGFR-TKIs.

**Table 10:** Advances of FGFR-TKIs

Drug	Brand name	Manufacturer	Targets	Applications of diseases	Approved years or current phase of clinical trials
Erdafitinib	Balversa	Janssen	FGFR1-4	FGFR2/3-alterated locally advanced or metastatic urothlial cancer that has progressed during or following platinum-containing chemotherapy	2019 [166]
Pemigatinib	Pemazyre	Incyte	FGFR1-3	Previously treated, unresectable locally advanced or metastatic CCA with FGFR2 fusion+ or other rearrangement	2020 [167]
Investigational drugs					
Futibatinib	-	Taiho Oncology	FGFR1-4	FGFR2-rearranged advanced intrahepatic CCA	III (NCT04093362)
				FGFR1-4 rearranged solid tumors	II (NCT04189445)
				FGFR-amplifiled MBC	II (NCT04024436)
Infigratinib	-	Novartis Oncology	FGFR1-3	previously treated advanced FGFR3-rearranged urothlial cancer previously treated advanced FGFR2-rearranged CCA	II [164] II [168]

In the last column of “Approval years or current phases of clinical trials”: erdafitinib and pemigatinib were firstly by Food and Drug Administration (FDA); other two drugs are under investigation, we provided current phases of their clinical trials

*CCA* cholangiocarcinoma, *MBC* metastatic breast cancer, *FGFR* fibroblast growth factor receptor

Data source: www.fda.gov, www.drugs.com, and www.clinicaltrials.gov (cutoff date: 19 July 2020)

### KIT

KIT proto-oncogene takes part in fertility, homeostasis, and melanogenesis, while the dysregulation of KIT has been found to participate in the occurrence of leukemia, gastrointestinal stromal tumor (GIST), melanoma, and other cancers [169]. KIT usually presents in multi-targeted TKIs as an inconspicuous target (Table 5) since a single selective KIT-TKI failed to cure most cancers. However, the aberrant activation of KIT is particularly responsible for the tumorigenesis of GIST, making it a pivotal target in this disease entity. Besides, KIT inhibition also showed efficacy in KIT-positive melanoma.

### PDGFR

Platelet-derived growth factor (PDGF) is a family of a multi-functional polypeptide involved in cellular growth, proliferation, differentiation, and angiogenesis. PDGFR is found to play a crucial role in angiogenesis by promoting the maturation of new blood vessels and up-regulating the expression of VEGF [170]. Most VEGFR-associated multi-kinase inhibitors target PDGFR as well to augment anti-angiogenesis effect and suppress tumor growth (Table 5). Additionally, the inhibition of PDGFR plays an important role specifically in the treatment of GIST (Table 11).

**Table 11:** Advances of KIT/PDGFR-TKIs

Drug	Brand name	Manufacturer	Targets	Applications of diseases	Approval year or current phase of clinical trials
Imatinib	Gleevec	Novartis Oncology	Bcr-Abl, KIT, PDGFR	Advanced Philadelphia chromosome positive chronic myeloid leukemia	2001
				1L KIT + unrectable or metastatic GIST	2002
				Other hematological cancer	
Sunitinib	Sutent	Pfizer Inc	KIT, PDGFR, VEGFR1-2, FLT3	GIST after disease progression on or intolerance to imatinib	2006 [172]
Regorafenib	Stivarga	Bayer	KIT, PDGFR, VEGFR1-3, TIE2, FGFR, BRAF, RET	Locally advanced, unresectable, or metastatic GIST previously treated with imatinib and sunitinib	2013 [173]
Avapritinib	Ayvakit	Blueprint Medicines	KIT, PDGFR	Unresectable or metastatic GIST harboring PDGFRA exon 18 mutation	2020 [176]
				≥ 4L and PDGFRA exon 18 mutant GIST	I [176]
				Locally advanced unresectable or metastatic GIST who previously received imatinib and 1 or 2 other TKIs	III (NCT03465722)
Ripretinib (DCC-2618)	Qinlock	Deciphera	KIT, PDGFR	Advanced GIST who have received prior treatment with 3 or more TKIs including imatinib	2020 [177]
				Advanced GIST after disease progression on or intolerance to imatinib	III (NCT03673501)
Investigational drugs					
PLX-9486	–	Plexxikon	KIT	Combined with pexidartinib for KIT + GIST	[179]

In the last column of “Approval years or current phases of clinical trials”: if a drug has been approved, we provided data of the year of its approval by Food and Drug Administration (FDA); if a drug is under investigation, we provided current phases of its clinical trials

*GIST* Gastrointestinal stromal tumor, *TKIs* tyrosine kinase inhibitors, *PDGFR* platelet-derived growth factor receptor

Data source: www.fda.gov, www.drugs.com, and www.clinicaltrials.gov (cutoff date: 19 July 2020)

### KIT- and PDGFR-TKIs in GIST

GIST generally resists to conventional chemotherapy. Fortunately, since GIST has high frequency of KIT and PDGFR mutation (approximately 80% of GISTs harbor KIT mutation, 10% involve PDGFR mutations), KIT and PDGFR inhibition has been recognized as the primary therapeutic modality for unresectable or metastatic GIST [171]. Table 8 summarizes advances of KIT/PDGFR TKIs.

Imatinib remains as first-line treatment of KIT-positive unresectable GIST. Though more than half of GISTs respond to imatinib, resistance inevitably occurs. Sunitinib and regorafenib are the standard second- and third-line treatment for advanced GIST, respectively [172, 173]. Sunitinib greatly improved median time to tumor progression than placebo (27.3 vs 6.3 weeks; HR 0.33; *p* < 0.0001) in patients with advanced GIST after failure of imatinib, but with a low ORR of 6.8% [174]. Studies indicated the inconsistent activity of sunitinib in imatinib-resistant populations, with higher response in patients harboring ATP-binding pocket mutations than those with mutations in KIT activation loop [175].

Recently, two selective TKIs targeting KIT and PDGFRA mutants, avapritinib and ripretinib, were approved as fourth-line treatment for GIST. A phase I trial of avapritinib demonstrated an ORR of 86% in GIST patients with PDGFRA exon18 mutation and an ORR of 22% in those who have failed ≥ third-line treatment. Toxicity was generally manageable with anemia, fatigue, hypophosphatemia, hyperbilirubinemia, neutropenia, and diarrhea being the most common grade 3–4 AEs [176]. A phase III trial of ripretinib demonstrated an improved mPFS (6.3 vs 1.0 months; HR 0.15; *p* < 0.0001) and mOS (15.1 vs 6.6 months; HR 0.36; *p* = 0.0004) compared to placebo [177, 178]. Besides, in a phase I study, an investigational KIT inhibitor PLX9486 alone or in combination with pexidartinib presented preliminary efficacy against resistant GIST [179].

### KIT inhibition in Melanoma

KIT mutations occur in 35–40% of mucosal and acral melanoma, and 28% of melanomas on chronically sun-damaged skin [180]. Imatinib and nilotinib demonstrated ORRs of 17–30% and disease control rates (DCRs) of 35–57% in metastatic melanoma patients with KIT mutation/amplification [181–183]. However, most of the reported response only had short duration, and no further phase III trials have been conducted. Until now, none KIT-TKI has received an approval for KIT-mutant melanoma.

## Other tyrosine kinase

The insulin-like growth factor 1 receptor (IGF-1R) is a RTK involved in the growth and survival of normal and neoplastic cells; however, multiple trials of IGF-1R inhibitors failed to show definitive clinical benefit [184]. For example, a phase III trial of IGF-1R inhibitor linsitinib for patients with advanced adrenal corticocarcinoma failed to prolong either PFS or OS compared to placebo [185].

Bruton's tyrosine kinase (BTK), an intracellular NRTK, plays a crucial role in B-cell antigen receptor (BCR) signaling pathway. The application of BTK inhibitors, such as ibrutinib, acalabrutinib, and zanubrutinib, is considered as a breakthrough in B-cell-related hematological malignancies and autoimmune diseases, but with limited positive finding in solid tumors [186]. Nevertheless, increasing knowledge of off-target effects of BTK inhibitors and B-cells’ role in proliferation of solid tumors still encourages further but careful exploration of BTK inhibitors in solid malignancies, either as single agent or in combination with other treatment strategies like ICIs [187, 188].

## Prospects and conclusions

Twenty years have passed since the approval of the pioneer TKI imatinib for chronic myeloid leukemia in 2001 which was deemed as the beginning of targeted therapeutic era. Increasing numbers of TKIs for tough-to-inhibit oncogenic targets are available for clinical use, providing precise targeted therapy options based on individual patients’ genetic alteration features. TKIs have dramatically improved patients’ survival and quality of life, and shifted cancer treatment paradigm.

Despite numerous advances, therapeutic responses of TKIs vary widely in individual patients and across patient populations, depending on multiple factors such as potency and selectivity of TKIs, variability of drug metabolism and pharmacokinetics profiles among individuals, tumor biology including tumor heterogeneity, and tumor microenvironment, etc.[189]. Drug resistance (de novo or acquired resistance) inevitably develops. Mechanisms of acquired resistance to TKIs could be generally categorized into three classes: i) on-target mutations, like EGFR-T790M to first-/second-generation EGFR-TKIs; ii) off-target mutations: parallel, downstream or alternative pathways activation, like EGFR-independent resistant mechanisms caused by MET/HER2 amplification, HGF overexpression, etc.; iii) histological transformation into another type such as neuroendocrine or mesenchymal tumor [190].

How to solve and prevent drug resistance will be the key issue for future TKIs development. Besides, to improve safety profile and patient’s compliance, it’s also critical to develop more selective TKIs since multi-targeted TKIs might cause unnecessary off-target toxicities by the inhibition of irrelevant targets. Next-generation TKIs are designed to overcome on-target resistance and serve as therapeutic options after progression of former-generation ones. They are generally equipped with enhanced therapeutic efficacy and selectivity, some with better penetration to BBB and CNS responses, and even are recommended or have potential to take place the standard of care. But for other off-target mechanisms, original compounds combined with targeted agents for newly discovered aberration might work. Besides, a series of clinical studies are exploring TKI combination treatment with antibodies or other inhibitors involving different mechanisms to amplify efficacy.

Future collaborative efforts are expected to enhance understanding of resistance mechanism of TKIs; to develop more potent, selective, and better BBB-penetrated TKIs or next-generation TKIs; and to discover more effective and low toxic combinational therapy and sequency. These attempts will help overcome resistance and bring further survival benefit and better quality of life for patients with solid tumors in the future.

## Data Availability

All data generated or analyzed during this study are included.

## Abbreviations

TKI: Tyrosine kinase inhibitors
ATP: Adenosine triphosphate
RTK: Receptor tyrosine kinases
NRTK: Non-receptor tyrosine kinases
EGFR: Epidermal growth factor receptor
ALK: Anaplastic lymphocyte kinase
HER2: Human epidermal growth factor receptor 2
TRK: Tropomyosin receptor kinase
VEGFR: Vascular endothelial growth factor receptor
RET: Rearranged during transfection
MET: Mesenchymal–epithelial transition factor
PDGFR: Platelet-derived growth factor receptor
Non-MSI-H: Non-microsatellite instability-high
FGFR: Fibroblast growth factor receptor
mPFS: Median progression-free survival
mOS: Median overall survival
USA: The United States of America
ORR: Overall response rate
DCR: Disease control rate
HR: Hazard ratio
AEs: Adverse events
TRAE: Treatment-related adverse events
FDA: Food and Drug Administration
NMPA: National Medical Products Administration
EMA: European Medicines Agency
CNS: Central nervous system
BBB: Blood–brain barrier
ILD: Interstitial lung disease
NSCLC: Non-small cell lung cancer
SqCC: Squamous cell carcinoma
MBC: Metastatic breast cancer
MHLW: Ministry of Health, Labor and Welfare
NTRK: Neurotrophin receptor tyrosine kinase
ECOG: Eastern Cooperative Oncology Group
PS: Performance status
ALT: Alanine aminotransferase
AST: Aspartate aminotransferase
PD-1: Programmed cell death protein-1
PD-L1: Programmed death ligand 1
ICIs: Immune checkpoint inhibitors
SCLC: Small cell lung cancer
mRCC: Metastatic renal cell carcinoma
HCC: Hepatocellular carcinoma
DTC: Differentiated thyroid cancer
MTC: Medullary thyroid cancer
CRC: Colorectal cancer
GIST: Gastrointestinal stromal tumor
STS: Soft tissue sarcoma
NET: Neuroendocrine tumors
NPC: Nasopharyngeal carcinoma
FLT3: Fetal liver tyrosine kinase receptor 3
RCC: Renal cell carcinoma
EC: Endometrial cancer
HGFR: Hepatocyte growth factor receptor
METex14: MET exon14 skipping alterations
PSC: Pulmonary sarcomatoid carcinoma
CCA: Cholangiocarcinoma
NDA: New drug application
IGF-1R: Insulin-like growth factor 1 receptor
BTK: Bruton's tyrosine kinase
BCR: B-cell antigen receptor
